# The serotonin transporter gene polymorphism and the effect of baseline on amygdala response to emotional faces

**DOI:** 10.1016/j.neuropsychologia.2010.12.013

**Published:** 2011-03

**Authors:** Elisabeth A.H. von dem Hagen, Luca Passamonti, Sarah Nutland, Jennifer Sambrook, Andrew J. Calder

**Affiliations:** a MRC Cognition and Brain Sciences Unit, 15 Chaucer Road, Cambridge CB2 7EF, UK; b Consiglio Nazionale delle Ricerche, Unità di Ricerca Neuroimmagini, Catanzaro, Italy; c Juvenile Diabetes Research Foundation/Wellcome Trust Diabetes and Inflammation Laboratory, Cambridge Institute for Medical Research, University of Cambridge, Cambridge CB2 0XY, UK; d Department of Haematology, University of Cambridge, Cambridge, UK; e National Health Service Blood and Transplant, Cambridge, UK

**Keywords:** Serotonin, Amygdala, Facial expressions, fMRI

## Abstract

Previous research has found that a common polymorphism in the serotonin transporter gene (5-HTTLPR) is an important mediator of individual differences in brain responses associated with emotional behaviour. In particular, relative to individuals homozygous for the l-allele, carriers of the s-allele display heightened amygdala activation to emotional compared to non-emotional stimuli. However, there is some debate as to whether this difference is driven by increased activation to emotional stimuli, resting baseline differences between the groups, or decreased activation to neutral stimuli. We performed functional imaging during an implicit facial expression processing task in which participants viewed angry, sad and neutral faces. In addition to neutral faces, we included two further baseline conditions, houses and fixation. We found increased amygdala activation in s-allele carriers relative to l-homozygotes in response to angry faces compared to neutral faces, houses and fixation. When comparing neutral faces to houses or fixation, we found no significant difference in amygdala response between the two groups. In addition, there was no significant difference between the groups in response to fixation when compared with a houses baseline. Overall, these results suggest that the increased amygdala response observed in s-allele carriers to emotional faces is primarily driven by an increased response to emotional faces rather than a decreased response to neutral faces or an increased resting baseline. The results are discussed in relation to the tonic and phasic hypotheses of 5-HTTLPR-mediated modulation of amygdala activity.

## Research highlights

▶ 5-HTTLPR short allele carriers have greater amygdala response to emotional stimuli. ▶ Increased amygdala activity in s-carriers is consistent regardless of baseline. ▶ The results support a largely phasic model of 5-HTTLPR-mediated amygdala modulation.

## 1 Introduction

Serotonin is a fundamental neuromodulator within brain regions involved in emotional behaviour such as the amygdala (Hariri & Holmes, 2006). Abnormalities in the serotonergic system have been found in individuals with mood, anxiety and aggressive disorders, and treatment of these psychiatric conditions often involves pharmacological manipulation of the serotonergic system (Ballenger, 1999; Blier & de Montigny, 1999). In recent years there has been considerable interest in determining possible genetic factors underlying the regulation of serotonin levels, and whether individual differences in temperament and vulnerability to emotional disorders might reflect variation in the expression of serotonergic genes. A candidate gene that has been studied extensively in this context is the serotonin transporter (5-HTT) gene (*SLC6A4*). Lesch et al. (1996) found that a common genetic variation in the transcription region (5-HTTLPR) of this gene, which consists of short- (s-) and long- (l-) allele variants, results in differential expression of 5-HTT, with the s-allele variant expressing less 5-HTT protein than the l-allele.

Individuals who carry at least one copy of the s-allele have been associated with having increased levels of anxiety, reduced capability to deal with stress, and thus greater susceptibility to depression in the context of stressful life events (Caspi et al., 2003; Caspi, Hariri, Holmes, Uher, & Moffitt, 2010). Furthermore, functional neuroimaging studies have consistently identified the amygdala as a key brain region where the s-allele genotype exerts its effects. Hariri et al. (2002) were the first to report that, relative to individuals homozygous for the l-allele, carriers of the s-allele displayed heightened amygdala activation to fearful and angry facial expressions compared with non-emotional stimuli, suggesting that the 5-HTT polymorphism is implicated in the modulation of affective behaviour through the amygdala. The differential activation of the amygdala to emotional stimuli in carriers of the s-allele has since been replicated in several independent studies using similar tasks (Munafo, Brown, & Hariri, 2008), as well as in studies using a variety of other types of emotional stimuli including negative words (Canli et al., 2005), aversive pictures (Heinz et al., 2005), and subliminally presented sad emotional facial expressions (Dannlowski et al., 2009). All of these studies have consistently reported differential amygdala response in the s-allele carriers relative to l-allele homozygotes.

Nonetheless, there is an active debate as to whether the difference in amygdala activation between s-carriers and l-homozygotes is driven by increased activation of the amygdala to the emotional stimuli themselves, an altered amygdala response at rest, or a decreased response to neutral stimuli. Since functional imaging studies are necessarily a contrast between two conditions, the selection of a ‘baseline’ impacts highly on the conclusions that can be drawn with respect to an observed effect. In their study of 5-HTTLPR genotype and its effect on neural function, Canli et al. (2005) suggested that the relative increase in amygdala activation to emotional stimuli in s-carriers compared to l-homozygotes was not due to hyperreactivity to emotional stimuli. Instead, they proposed that it was driven by a decreased activation to neutral stimuli or an increased response to the fixation condition in the s-carriers. Their observations were based on contrasts with a fixation baseline, where s-carriers displayed reduced activation to neutral stimuli compared with fixation. A follow-up study examining the effects of gene by environment interactions as a function of serotonin transporter genotype using perfusion imaging suggested that this effect was actually driven by an increase in ‘rest’ activity in s-carriers (Canli et al., 2006).

To further address this issue, Heinz et al. (2007) reanalysed the data from their previous study (Heinz et al., 2005) with respect to fixation. They found a similar reduction in response to neutral stimuli relative to fixation. However, they suggested that while this resulted from an increased response in s-carriers to the fixation condition, the effect should be ascribed to the heightened anxiety associated with the ambiguity and uncertainty of a fixation condition. Thus, their interpretation would be in keeping with an increased s-carrier response to negative or anxiogenic stimuli. Both Canli et al. (2005) and Heinz et al. (2007) however had only neutral and fixation baseline conditions so the impact of fixation could not be assessed by contrasting it with a third independent baseline.

**Fig. 1:**
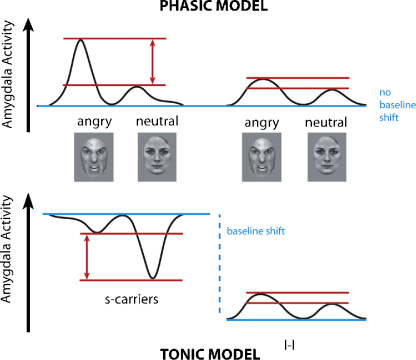
Schematic depiction of hypothesized amygdala activity in response to angry and neutral facial expressions, as predicted by Canli & Lesch (2007), for the phasic and tonic models of 5-HTTLPR-mediated amygdala responsivity. In the phasic model, both s-carriers and l-homozygotes have a similar baseline and stimuli cause an increase in amygdala activity. In the tonic model, the baseline for s-carriers has been shifted up such that amygdala response to angry and neutral stimuli is now reflected as a decrease with respect to the elevated baseline.

In light of these interpretations of 5-HTTLPR genotype action, Canli and Lesch (2007) put forward two models of 5-HTT modulation of amygdala activation (Fig. 1). The phasic model proposes that the presence of the s-allele results in increased amygdala reactivity to emotional stimuli, whereas the tonic model proposes that the differential amygdala activation in s-carriers and l-homozygotes is driven by a baseline difference between the two groups such that s-allele carriers display heightened amygdala activity compared to non-carriers at rest (see Fig. 1). While Heinz et al.’s (2007) interpret their data in terms of the phasic model, Canli et al.’s results (2005, 2006) support a primarily tonic model, but it is important to note that the two models are not necessarily mutually exclusive. Two further studies have since addressed the issue of a proposed tonic baseline shift by examining the influence of 5-HTTLPR on resting state activity and found increased perfusion of the amygdala in s-carriers, providing support for a tonic baseline shift (Canli et al., 2006; Rao et al., 2007). However, a more recent study, the largest to date with 183 subjects, found no association between resting blood perfusion in the amygdala and 5-HTTLPR genotype (Viviani et al., 2010).

Here, we address these models by performing functional imaging during an implicit negative emotional facial expression processing task with three ‘baseline’ conditions: neutral facial expressions, houses and fixation. We chose to use facial expressions as a stimulus set because many studies have used facial expressions in conjunction with the study of 5-HTTLPR, but the studies discussing the tonic/phasic models have primarily used emotional pictures or words (Canli et al., 2005; Heinz et al., 2007). Support for the phasic model of the 5-HTTLPR polymorphism's mechanism of action would require an increased amygdala response in s-carriers to negative emotional expressions regardless of baseline, with the possible exception of fixation if indeed it is an anxiogenic stimulus as suggested by Heinz et al. (2007). Support for the tonic model would require elevated baseline amygdala activity in s-carriers which may manifest as a significant reduction in amygdala activation for comparisons of neutral faces or houses with a fixation baseline and no significant difference in response between negative facial expressions and fixation (see Fig. 1).

## 2 Methods

### 2.1 Subjects

Sixty-eight healthy participants (23 male) with age range 20–41 years (mean = 31 ± 6) participated in the study. Participants were recruited from the Cambridge BioResource, a large panel of volunteers that agreed to take part in research linking genotype with phenotype (http://www.cambridgebioresource.org.uk/). All volunteers were right-handed European or North American Caucasians. Participants were subdivided into two groups based on their 5-HTTLPR genotype, with one group consisting of carriers of either one or two s-alleles (*n* = 45, mean age = 30 ± 6, 17 male) and the other group consisting of l-allele homozygotes (*n* = 23, mean age = 31 ± 6, 12 male). This grouping was based on previous observations that lymphoblasts of carriers of either one or two copies of the s-allele had similar uptake levels of 5-HTT, whereas l-allele homozygotes had twofold higher 5-HTT uptake (Lesch et al., 1996). Previous functional imaging studies have also grouped homozygous and heterozygous s-carriers together (Hariri et al., 2002; Passamonti et al., 2008). The two groups did not significantly differ in age (*t*(61) = 0.22, *p* = 0.83) or gender (*χ*^2^(1,63) = 0.32, *p* = 0.57). Participants’ mental and physical health was screened prior to genotyping using a detailed medical history questionnaire used by the Cambridge BioResource. This revealed no history of neurological disease or psychiatric disorders, and no participants were on any medication affecting the central nervous system. All participants had normal or corrected to normal vision, and no structural brain abnormalities were detected in their MRI scans. The study was approved by the Cambridgeshire Local Research Ethics Committee, and all participants gave their informed written consent. Participants also completed the Spielberger State-Trait Anxiety Inventory (STAI) (Spielberger, 1983) and had scores ranging from 20 to 49 (mean 32 ± 7) for the State Anxiety scale and from 20 to 58 (mean 37 ± 9) for the Trait Anxiety scale. Three participants neglected to fully complete the Trait Anxiety questionnaire but were nevertheless included in the imaging analysis. Neither the State Anxiety nor the Trait Anxiety scores differed significantly between the two genotype groups (*t*(58) = 0.22, *p* = 0.73 and *t*(61) = 0.35, *p* = 0.83, respectively).

### 2.2 Genotyping

All subjects were genotyped for 5-HTTLPR. Samples were genotyped using the Taqman 5 nuclease assay (Applied Biosystems, Warrington, UK) according to the manufacturer's protocol. DNA was extracted from blood samples obtained from all subjects according to standard procedures. The 5-HTT regulatory gene region was amplified by polymerase chain reaction (PCR) with the following oligonucleotide primers: forward 5′-GGCGTTGCCGCTCTGAATGC-FAM -3′; reverse 5′-GAGGGACTGAGCTGGACAACCAC-3′. PCR was performed with a fluorescent label for each sample and the fragment size measured on an ABI3730xl DNA Analyser and scored using ABI genemapper software. To minimise error, two operators independently scored the genotypes. The allele size and numbers of repeat sequences observed were scored as follows: 485–16 Repeat (−44 bp) (short); 529–16 Repeat (long). Allele frequencies were 58% for the long and 42% for the short allele variant, and genotype frequencies did not deviate from the Hardy–Weinberg equilibrium with 34% l-l, 48% s-l, and 18% s-s (*χ*^2^ < 0.004, *p* = 0.97, df = 1).

All subjects were also genotyped for catechol-*O*-methyl transferase (COMT), since previous research found that a polymorphism in the COMT gene (*val*^158^*met*) modulates amygdala response to emotional stimuli (Smolka et al., 2005). The *val*^158^*met* COMT polymorphism was assayed by PCR amplification using the following primers: forward 5′-CCCAGCGGATGGTGGAT-3′ and reverse 5′-CAGGCATGCACACCTTGTC-3′. The amplification conditions were initiated at 95 °C for 10 min, followed by 40 cycles consisting of denaturation at 92 °C for 15 s, and annealing and extension at 60 °C for 1 min. 24 participants were homozygous for the *met*^158^ allele, 20 participants were homozygous for the *val*^158^ allele, and 24 participants were heterozygotes. There was no significant association between the 5-HTT*LPR* and COMT genotypes (*χ*^2^ = 3.46, df = 4, *p* = 0.50).

### 2.3 Stimuli

Facial stimuli consisted of grey-scale images with angry, sad and neutral expressions selected from the NimStim Face Stimulus Set (Tottenham et al., 2009) and the Karolinska directed emotional faces (KDEF) (Lundqvist & Litton, 1998) set based on independent emotional ratings. There were 30 different identities (half female) for each emotional expression. House stimuli consisted of grey-scale images of two- or three-storey homes, all viewed from the front of the building. Stimuli were presented in a blocked design comprising eight 17.6s blocks of each of four conditions: angry, sad, and neutral facial expressions, and houses. Each block consisted of 4 stimuli presented for 4s with an ISI of 400 ms. Participants were instructed to respond by button press whether the stimulus was shifted by 0.5° to the left or right of the centre of the screen. Each stimulus block was followed by 8s of fixation, where participants were instructed to passively view the fixation cross at the centre of the screen. Gender and identity of the faces were fully randomized throughout the experiment, and block order was presented in one of two pseudorandom orders. Reaction times and accuracy were recorded. Total experiment duration was 13 min and 40 s.

### 2.4 Functional imaging data acquisition and analysis

Whole-brain T2*-weighted echo-planar-imaging (EPI) was performed on a Siemens 3T Tim Trio (32 slices, TR = 2 s, TE = 30ms, voxel size 3 mm × 3 mm × 3 mm) at the MRC Cognition & Brain Sciences Unit. The first 4 scans were discarded to allow for equilibration effects. Magnetization-prepared rapid-acquisition gradient echo T1-weighted structural scans were also acquired in all subjects with 1 mm × 1 mm × 1 mm resolution.

Imaging data were analysed using SPM5 (http://www.fil.ion.ucl.ac.uk/spm). EPI scans were corrected for slice timing differences and realigned to the first functional scan using rigid-body transformations to correct for head movement. EPI and structural images were coregistered and normalized to the T1 standard template in MNI space (Montreal Neurological Institute (MNI)—International Consortium for Brain Mapping) using linear and non-linear transformations, and smoothed with a Gaussian kernel of full-width-half-maximum (FWHM) 8 mm. Six participants (3 s-carriers, 3 l-homozygotes) were removed from the analysis following pre-processing due to gross movement during the scanning session (>2 mm) (two participants) or signal dropout in the medial temporal lobes (four participants).

Data were analysed using the general linear model as implemented in SPM5. Condition effects were modelled using boxcar regressors convolved with a canonical hemodynamic response function. Spatial realignment parameters were included as regressors of no interest in the model to account for residual movement-related variance. A high-pass filter at 128 s removed low-frequency signal drifts. Statistical parametric maps were generated for each individual by estimating activation contrasts between all conditions (e.g. angry vs. neutral faces, sad faces vs. houses).

For each subject, data were extracted from the left and right amygdala using an anatomical *a priori* amygdala region-of-interest derived from the Anatomical Automatic Labelling (AAL) software (Tzourio-Mazoyer et al., 2002). Group differences in the amygdala response to emotional expressions were determined in SPSS (SPSS Inc., Chicago, IL) with a repeated measures ANOVA in a group (s-carrier and l-homozygotes; between subjects) by hemisphere (left and right; repeated measure) by emotion (angry and sad; repeated measure) design. Separate ANOVAs were conducted for each of the 3 baseline conditions (e.g. emotion vs. neutral, emotion vs. houses, emotion vs. fixation). In addition, group differences across the baseline conditions were determined by contrasting the neutral facial expression condition with a fixation or houses baseline, and contrasting fixation with a houses baseline in three separate group by hemisphere repeated measure ANOVAs. Adjustment for multiple comparisons was performed by Bonferroni correction and significance levels determined at *p* < 0.05. Within each group, contrast estimates that were significantly different from zero were determined by one-sample *t*-tests.

## 3 Results

### 3.1 Behavioural results

There was no significant difference between the s-carriers and l-homozygotes in their ability to indicate the position of the stimuli on the screen. Both reaction times and accuracy (measured as percent correct responses) were not significantly different (reaction times mean ± SD: s-carriers = 798 ± 29 ms, l-l = 815 ± 43 ms, *F*(1,60) = 0.11, *p* = 0.74; percent correct: s-carriers = 97 ± 5%, l-l = 96 ± 6%, *F*(1,60) = 0.65, *p* = 0.43).

### 3.2 Functional imaging results

**Fig. 2:**
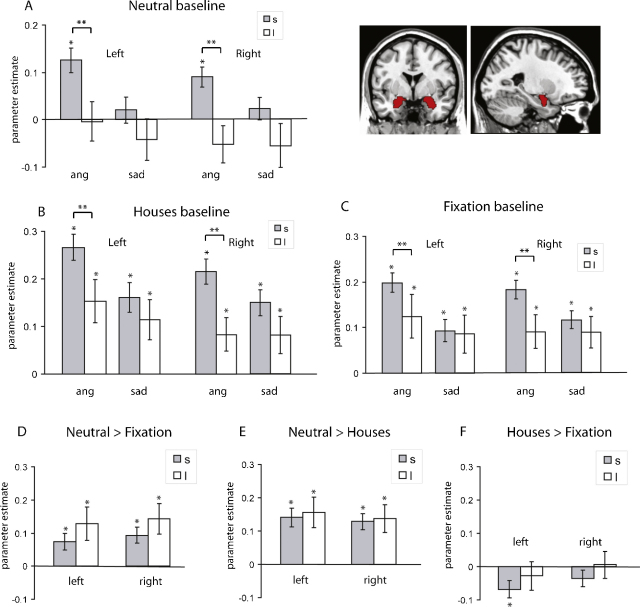
Extracted amygdala response from left and right hemisphere AAL regions-of-interest for s-allele carriers (grey shaded bars) and l-allele homozygotes (white bars) for angry and sad facial expressions compared to (A) a neutral facial expressions baseline, (B) a houses baseline, and (C) a fixation baseline. Contrasts across the baseline conditions are shown in (D) for neutral facial expressions compared to fixation, (E) for neutral facial expressions compared to houses, and (F) for houses compared to fixation. A ** represents a significant (*p* < 0.05) pairwise corrected group difference. A * indicates a parameter estimate significantly (*p* < 0.05) greater than 0. Error bars represent the standard error of the mean. Coronal and sagittal sections depict the extent of the AAL regions-of-interest from which data were extracted.

The extracted amygdala responses for the group analyses are displayed in Fig. 2.

#### 3.2.1 Emotion vs. neutral baseline

We found a significant difference in bilateral amygdala activation between carriers of the s-allele and l-allele homozygotes in response to angry and sad facial expressions compared to neutral facial expressions (*F*(1,60) = 6.77, *p* = 0.01), with s-carriers displaying a greater response to emotional compared to neutral faces than l-homozygotes (Fig. 2a). There was no significant group by hemisphere interaction (*F*(1,60) = 0.30, *p* = 0.59), however there was a borderline group by emotion interaction (*F*(1,60) = 2.91, *p* = 0.09). Despite the fact that the latter effect was not significant, we broke down this interaction to determine whether the difference in amygdala response to emotion between the groups was more pronounced for angry faces than for sad faces. Consistent with this hypothesis, a pairwise corrected comparison revealed that there was a significant difference between the groups for the angry faces condition (*p* = 0.002) but not for the sad faces (*p* = 0.13). Irrespective of group, there was a significantly greater amygdala response for the comparison angry vs. neutral than sad vs. neutral facial expressions (*F*(1,60) = 7.77, *p* = 0.007). There was also a significant emotion by hemisphere interaction (*F*(1,60) = 5.46, *p* = 0.02), reflecting a significantly greater response in the left hemisphere for angry vs. neutral facial expressions but not for sad vs. neutral expressions.

#### 3.2.2 Emotion vs. houses baseline

When emotional facial expressions were contrasted with a houses baseline (Fig. 2b), s-carriers again displayed significantly greater response to emotional facial expressions than the l-homozygotes (*F*(1,60) = 4.39, *p* = 0.04). Similar to the contrasts with a neutral facial expression baseline, there was no significant group by hemisphere interaction (*F*(1,60) = 0.59, *p* = 0.45), but there was a borderline group by emotion interaction (*F*(1,60) = 2.92, *p* = 0.09). A pairwise comparison to break down this borderline interaction again revealed that there was a significant difference between the two groups for the angry facial expression condition (*p* = 0.009) but not for the sad facial expression condition (*p* = 0.24). Irrespective of group, there was a significantly greater amygdala response for the comparison angry faces vs. houses than sad faces vs. houses (*F*(1,60) = 7.77, *p* < 0.007). There was also a significant emotion by hemisphere interaction (*F*(1,60) = 5.46, *p* = 0.02), reflecting a significantly greater response in the left hemisphere for angry facial expressions vs. houses but not for sad expressions vs. houses.

#### 3.2.3 Emotion vs. fixation baseline

When the groups’ amygdala response to emotional facial expressions were contrasted with a fixation baseline (Fig. 2c), there was no significant group difference (*F*(1,60) = 2.08, *p* = 0.16) between s-carriers and l-homozygotes. However, again we wanted to investigate the effect of group on angry and sad faces separately since we found a borderline group by emotion interaction (*F*(1,60) = 2.91, *p* = 0.09). Pairwise corrected comparisons revealed a significant group difference for the angry facial expression condition (*p* = 0.04) but not for the sad facial expression condition (*p* = 0.66). Irrespective of group, there was a significantly greater amygdala response for the angry than for the sad facial expressions contrasted with fixation (*F*(1,60) = 7.77, *p* = 0.007). The hemisphere by emotion interaction also achieved significance (*F*(1,60) = 5.46, *p* = 0.02), again reflecting a greater response in the left hemisphere for angry facial expressions. There was no interaction between group and hemisphere (*F*(1,60) = 0.59, *p* = 0.45).

#### 3.2.4 Angry facial expressions only

When the difference between angry faces and each of the three baseline conditions was compared across group in a separate repeated measures ANOVA, there was a significant group difference across all conditions (*F*(1,60) = 11.88, *p* = 0.001) indicating that there was a significantly greater response in s-carriers to angry faces regardless of baseline. Critically, there was no significant group by baseline interaction (*F*(1 = 2,59) = 0.69, *p* = 0.50), indicating that the magnitude of the difference between angry faces and each of the three baselines did not significantly differ.

#### 3.2.5 Baselines only

In order to address the tonic hypothesis, we compared amygdala response across the different ‘baseline’ conditions. A comparison of amygdala activation in the two groups for neutral facial expressions vs. fixation baseline (*F*(1,60) = 1.20, *p* = 0.28) (Fig. 2d), and houses vs. fixation baseline (Fig. 2f; *F*(1,60) = 0.81, *p* = 0.37) showed no significant difference between groups. Similarly, a group comparison for the contrasts neutral facial expressions compared to houses (Fig. 2e; *F*(1,60) = 0.06, *p* = 0.81) was also not significant.

#### 3.2.6 Additional analyses

Since previous research has identified a polymorphism in the COMT gene (*val*^158^*met*) as modulating amygdala response to emotional stimuli (Smolka et al., 2005), all of our participants were also genotyped for COMT and all analyses were repeated factoring out COMT genotype. All group effects reported above remained significant (*p*'s < 0.05).

Although age and gender did not differ significantly between groups, we repeated all analyses with age and gender as covariates in the ANOVAs. The outcome of the main effects remained similar. Full statistics are included in Supplemental materials.

Despite the small number of s-allele homozygotes (12 participants), we also looked at the effects of allele load by breaking down our analysis into three groups: s-homozygotes (s-s), l-homozygotes (l-l) and l-heterozygotes (s-l). Full details and statistics for this analysis are found in Supplemental materials. Results should be interpreted with caution however, as they may be underpowered due to the small s-homozygote group.

Finally, we also performed a regression analysis between Spielberger State and Trait Anxiety scores and amygdala activation for all contrasts. We found no significant association between amygdala activation in s-carriers or l-homozygotes across any of the contrasts or baseline comparisons.

## 4 Discussion

We have shown that carriers of the s-allele variant of 5-HTTLPR display heightened amygdala response relative to l-allele homozygotes in response to emotional facial expressions. These results therefore present further support for an effect of 5-HTTLPR on amygdala function. In addition, through the use of three baseline conditions, a houses, a fixation, and a neutral facial expressions condition, we found that the difference in amygdala response between the groups is primarily driven by increased differential activity of the amygdala to emotional stimuli rather than through altered amygdala response during neutral stimulus conditions or a baseline resting difference in amygdala activity between the groups.

While both Canli et al. (2005) and Heinz et al. (2007) found reduced amygdala activation in s-carriers to neutral words and pictures compared to fixation, we did not observe a significant reduction in s-carriers’ amygdala response to neutral facial expressions. In fact, both groups displayed a statistically similar increase in amygdala activation to neutral faces compared with both fixation and a houses baseline (Fig. 2d and e). A key difference that distinguishes our study from the previous studies addressing the tonic-phasic hypotheses is our use of facial expressions as a stimulus set. Single-cell recording has shown that the amygdala contains face-responsive cells (Gothard, Battaglia, Erickson, Spitler, & Amaral, 2007; Leonard, Rolls, Wilson, & Baylis, 1985) and this may explain why even neutral facial expressions engaged the amygdala, an observation consistent with previous research (Blasi et al., 2009; Schwartz et al., 2003; Wright et al., 2003). Importantly however, we observed no significant difference between the groups in their response to neutral facial expressions or houses relative to fixation. This is in contrast to the tonic model which predicts that the posited raised amygdala activity during rest (i.e., fixation) in the s-carriers should result in a significant group difference for neutral faces or houses in relation to fixation.

Although Canli et al. (2006) used facial expressions as a stimulus set in their gene by environment study, they did not report the group contrasts alone but only their interaction with the environment, so it is unclear whether or not they found an increased or decreased response in the amygdala in s-carriers in response to neutral faces compared with fixation. Canli et al. (2005) used affective words and Heinz et al. (2007) used affective pictures, but, despite observing the same effect in both of their studies when comparing fixation with neutral stimuli, their respective interpretations were different. While Heinz et al.’s (2007) interpretation is in line with a phasic model of amygdala activation to negative or anxiogenic stimuli, including a fixation condition, Canli et al. (2006) and Canli & Lesch (2007) interpret the results as a tonic resting amygdala activation difference between groups.

Our results provide greater support for a phasic model of amygdala activation as a result of 5-HTTLPR genotype since we observed significantly increased amygdala response in s-carriers to emotional facial expressions when compared with both a neutral faces and a houses baseline condition. Although the group difference for the contrast comparing emotional faces (angry and sad) with fixation did not quite reach significance, further exploration of the data showed significantly increased amygdala activation in s-carriers for angry faces vs. fixation. In addition, we found no significant difference between the change in BOLD response between viewing angry faces and each of the three baseline conditions (neutral faces, houses, and fixation), suggesting that the magnitude of the change BOLD response to angry faces was not significantly modulated by baseline. Consistent with this, when the baselines were directly contrasted with each other, we also found no significant difference between groups. Hence, we found no explicit support for the tonic model in which the action of 5-HTTLPR on the amygdala is driven by a passive resting baseline difference between the two groups (Canli et al., 2006). Nonetheless, we do not wish to exclude a ‘tonic contribution’ to our current data, particularly since the group difference between emotional faces and fixation was less evident than found relative to the neutral and house baselines. However, as suggested by Heinz et al. (2007) this may reflect the anxiogenic nature of the fixation condition rather than a raised baseline per se.

Determining resting baseline differences however when contrasting fixation with other conditions is contentious since activity at ‘rest’ is not independently assessed by this sort of analysis but is inferred with respect to another condition. To determine the presence of possible differences in resting baseline amygdala activity underlying the tonic model, three studies have looked at resting blood perfusion in the amygdala. As already discussed, two reported an association between s-carriers and relatively increased perfusion of the amygdala (Canli et al., 2006; Rao et al., 2007), lending credence to the tonic hypothesis. However, a recent study by Viviani et al. (2010), which looked at resting blood perfusion in a large cohort of 183 individuals genotyped for 5-HTTLPR, found no evidence for an effect of genotype on resting amygdala perfusion. Further studies will have to address resting amygdala differences to resolve the discrepancy in these results. It is also important to point out that the tonic and phasic models are not mutually exclusive, and that phasic activation to negative stimuli in s-carriers could occur in addition to a high baseline tonic activation. Our current results provide strong support for the phasic 5-HTTLPR-mediated amygdala activation, where emotional stimuli cause brief bursts of increased activation. Whether phasic-mediated effects might ride on top of a tonic-mediated raised baseline under certain conditions remains to be established, for instance in the case of a group of particularly high anxious s-carriers or in the case of gene by environment interactions. Our current data however provide evidence for a primarily phasic mediated response.

While the facial expression conditions and the houses condition all had a task associated with them, the fixation condition consisted of passive viewing of the central fixation cross. Thus, we might have expected the slightly stressful scanner environment to be brought to the forefront during the fixation condition and to preferentially raise anxiety levels to some degree in carriers of the s-allele as suggested by Heinz et al. (2007) – in particular because our participants were all novel to the scanner environment. However, although our results support a largely phasic model of amygdala activation, we found no significant evidence for an effect of heightened anxiety associated with the fixation condition.

While the phasic model provides an interpretation for the observed amygdala response associated with 5-HTTLPR, it is still unclear exactly how this effect is mediated at the level of 5-HTT action itself. Although research has suggested that a negative association exists between amygdala response during an emotion recognition task and 5-HTT availability in the amygdala, consistent with the putative action of 5-HTTLPR, the polymorphism itself was not assessed (Rhodes et al., 2007). Furthermore, a number of studies have failed to identify a significant relationship between in vivo binding of 5-HTT in the brain and s-allele carrier status (Parsey et al., 2006; Shioe et al., 2003; Willeit et al., 2001), and two recent studies in rhesus monkeys looking specifically at the availability of serotonin transporter in the amygdala found no differences between s-allele carriers and l-homozygotes (Christian et al., 2009; Jedema et al., 2010). It seems therefore that while 5-HTTLPR clearly modulates amygdala responsivity to emotional stimuli, it may not do so through direct action of 5-HTT in the amygdala itself. Future research is required to fully elucidate the mechanisms underlying amygdala hyperreactivity resulting from genetic variation in the serotonin transporter gene.

It is worth noting that most previous studies of 5-HTTLPR action on emotion processing in the amygdala have found a right lateralized effect (Canli et al., 2005; Hariri et al., 2002; Heinz et al., 2005), although this laterality was not explicitly addressed in these studies by comparing the left and right hemispheres. By contrast, we have observed bilateral amygdala response to emotional faces but no significant interaction between group and hemisphere.

Finally, there are certain limitations to our study which should be taken into consideration. Importantly, recent research has suggested that a third functional allele, labelled L_G_, a single-nucleotide polymorphism on the l-allele, is equivalent in expression to the s-allele and may modulate effects of 5-HTTLPR at a biological and/or behavioural level (Hu et al., 2005; Zalsman et al., 2006). As a result, it is possible that our results may be affected by a further breakdown of l-allele carriers into low and high expressing groups. However the allelic frequency of L_G_ in Caucasians is quite low (0.09–0.14) (Hu et al., 2005) and would therefore likely only represent a very small fraction of our participants. One further limitation relates to our experimental design which was a pseudo-randomization of experimental blocks with static fixation blocks following every experimental condition, hence increasing the predictability of the fixation condition. However, we chose this design deliberately to keep our design as similar as possible to Heinz et al.’s (2007) design, but increased the number of fixation blocks relative to Canli et al.’s (2005) study to increase the power.

### 4.1 Conclusions

In conclusion, we have shown that s-allele carriers display a heightened amygdala response to negative facial expressions and that this effect is driven primarily by an increased response to emotional facial expressions rather than a decreased response to neutral faces or an elevated resting baseline alone. These results provide further support for 5-HTTLPR-mediated amygdala modulation and suggest that amygdala response to emotional faces in s-carriers follows a phasic model of activation.
