# Supplementary material for: The serotonin transporter gene polymorphism and the effect of baseline on amygdala response to emotional faces
Source: Neuropsychologia. 2011 Mar;49(4):674–80. doi: 10.1016/j.neuropsychologia.2010.12.013 (PMC3209561; doi:10.1016/j.neuropsychologia.2010.12.013)
Supplement: Supplementary file 1 [file mmc1.doc]

**Supplemental Material**

**Effects of age and gender**

Full statistics for all ANOVAs with age and gender included as covariates-of-no-interest are detailed below.

*Emotion vs Neutral baseline*

There was a significant difference in bilateral amygdala activation between carriers of the s-allele and l-allele homozygotes in response to angry and sad facial expressions compared to neutral facial expressions (F(1,58)=6.838, p=0.01), with s-carriers displaying a greater response to emotional compared to neutral faces than l-homozygotes. There was no significant group by hemisphere interaction (F(1,58)=0.36, p=0.55), however there was a borderline group by emotion interaction (F(1,58)=2.63, p=0.11). A pairwise corrected comparison revealed that there was a significant difference between the groups for the angry faces condition (p=0.003) but not for the sad faces (p=0.11). Irrespective of group or emotion, there was a significantly greater amygdala response in the left hemisphere than in the right hemisphere (F(1,58)=5.14, p=0.03).

*Emotion vs Houses baseline*

When emotional facial expressions were contrasted with a houses baseline, s-carriers displayed significantly greater response to emotional facial expressions than the l-homozygotes (F(1,58)=4.15, p<0.05). There was no significant group by hemisphere interaction (F(1,58)=0.76, p=0.39), but there was a borderline group by emotion interaction (F(1,58)=2.64, p=0.11). A pairwise comparison to break down this borderline interaction again revealed that there was a significant difference between the two groups for the angry facial expression condition (p=0.01) but not for the sad facial expression condition (p=0.24). There was also a significantly greater response in the left hemisphere than in the right hemisphere irrespective of emotion and group (F(1,58)=13.72, p<0.001).

*Emotion vs Fixation baseline*

When the groups’ amygdala response to emotional facial expressions were contrasted with a fixation baseline, there was no significant group difference (F(1,58)=2.01, p=0.16) between s-carriers and l-homozygotes. Again, there was a borderline group by emotion interaction (F(1,58)=2.63, p=0.11). Pairwise corrected comparisons revealed a significant group difference for the angry facial expression condition (p=0.05) but not for the sad facial expression condition (p=0.62). There was no interaction between group and hemisphere (F(1,58)=0.57, p=0.46), neither was there a significant effect of hemisphere (F(1,58)=2.39, p=0.13).

*Angry facial expressions only*

When the groups were compared in their response to angry faces only across the three baseline conditions in a separate repeated measures ANOVA, there was a significant group difference across all conditions (F(1,58)=11.26, p=0.001) indicating that there was a significantly greater response in s-carriers to angry faces regardless of baseline. There was no significant group by baseline interaction (F(2,57)=0.70, p=0.50), but irrespective of group there was a significant effect of hemisphere (F(1,58)=4.57, p=0.04) and a significant effect of baseline (F(2,57)=3.61, p=0.03).

*Baselines only*

When we compared amygdala response across the different ‘baseline’ conditions, there was no significant difference between groups (F(1,58)=1.25, p=0.27) for the comparison of neutral facial expressions and fixation. Similarly, a group comparison for the contrast neutral facial expressions compared to houses (F(1,58)=0.10, p=0.75), and fixation compared to houses (F(1,58)=0.72, p=0.40) was also not significant.

**Effects of allele load**

Full statistics for all ANOVAs with data split into three groups according to allele load are detailed below. Group sizes were 20 long allele homozygotes (l-l), 30 long allele heterozygotes (s-l), and 12 short allele homozygotes (s-s). Due to the small size of the s-s group, the results should be interpreted with caution.

*Emotion vs Neutral baseline*

There was a significant group effect in response to angry and sad facial expressions compared to neutral facial expressions (F(2,59)=3.96, p=0.02). Pairwise comparisons revealed a significant difference between the l-l and s-s groups (p=0.03), a trend towards significance for l-l compared with s-l (p=0.12) and a non-significant difference between s-l and s-s (p=0.87). As reported in the two group analysis (s-carriers vs l-homozygotes), there was a main effect of emotion (F(1,59)=9.29, p<0.01) with angry facial expressions eliciting a greater amygdala response, and a significant hemisphere by emotion interaction (F(1,59)=5.95, p=0.02). Although there was no significant group by emotion interaction (F(2,59)=1.79, p=0.18), for consistency with our two group analysis we again broke down the interaction and found that there was a significant difference between the l-l and the s-l groups (p=0.02), and between l-l and s-s groups (p=0.02) for the angry facial expression condition but not for the sad facial expression condition (p’s > 0.15). There was no significant difference between the s-l and s-s groups for angry (p=1.0) or sad (p=0.63) facial expressions compared with a neutral baseline.

*Emotion vs Houses baseline*

For the comparison of angry and sad facial expressions with a houses baseline, there was a significant group difference (F(2,59)=4.42, p=0.02). Pairwise comparisons revealed a significant difference between the l-l and s-s groups (p=0.01), a trend towards significance for l-l compared with s-l (p=0.13) and a non-significant difference between s-l and s-s (p=0.57). In addition, there was a main effect of emotion (F(1,59)=9.29, p<0.01), a main effect of hemisphere (F(1,59)=7.01, p=0.01), and a hemisphere by emotion interaction (F(1,59)=5.95, p=0.02). Again, we broke down the interaction of group by emotion (F(2,59)=1.79, p=0.18) and found a significant difference between the s-s and l-l groups for the angry facial expression condition (p=0.01) and a borderline significant difference for the sad facial expression condition (p=0.07). We found no significant difference between the s-s and s-l groups nor between s-l and l-l for angry or sad facial expressions (p’s > 0.1).

*Emotion vs Fixation baseline*

When angry and sad facial expressions were compared with a fixation baseline between groups, we found no significant effect of group (F(2,59)=2.04, p=0.14). However, again we found a significant effect of emotion (F(1,59)=9.29, p<0.01), and a significant hemisphere by emotion interaction (F(1,59)=5.95, p=0.02). When we broke down the group by emotion interaction (F(2,59)=1.79, p=0.18), we found no significant differences between any of the groups for angry or sad facial expressions (p’s > 0.13).

*Angry facial expressions only*

For consistency with the analyses performed in the main body of the paper, we also compared the groups in their amygdala response to angry faces only relative to each of the three baseline conditions in a separate repeated measures ANOVA. There was a main effect of group across all baselines (F(2,59)=6.89, p<0.01) where pairwise comparisons revealed a significantly greater response in the s-s and s-l groups compared to l homozygotes (p’s 0.003 and 0.02 respectively) regardless of baseline, but no difference between the s-s and s-l groups (p=0.57). There was no significant group by baseline interaction (F(4,118)=0.60, p=0.66), but irrespective of group there was a significant effect of hemisphere (F(1,59)=8.23, p<0.01), a significant effect of baseline (F(2,58)=20.2, p<0.01), and a significant hemisphere by baseline interaction (F(2,58)=3.55, p=0.03).

*Baselines only*

When we compared amygdala response between different ‘baseline’ conditions (neutral vs fixation, neutral vs houses, and fixation vs houses), there was no significant effect of group for the comparison of neutral facial expressions and fixation (F(2,59)=0.6, p=0.55). Similarly, there was no significant effect of group for the contrast neutral facial expressions compared to houses (F(2,59)=0.47, p=0.63), neither was there a significant effect for fixation compared to houses (F(2,59)=0.75, p=0.48).
